# Highly Stretchable Conductive Hydrogel-Based Flexible Triboelectric Nanogenerators for Ultrasensitive Tactile Sensing

**DOI:** 10.3390/polym17030342

**Published:** 2025-01-26

**Authors:** Shan Huang, Weibin Wang, Chao Yang, Jianguo Liu, Kangshuai Li, Lina Zhou, Hao Zhang, Dongzhi Zhang

**Affiliations:** 1 College of Pipeline and Civil Engineering, China University of Petroleum (East China), Qingdao 266580, China; huangshan.slyt@sinopec.com; 2 Technology Inspection Center of Shengli Oilfield, China Petroleum & Chemical Corporation, Dongying 257000, China; wangweibin572.slyt@sinopec.com; 3 State Key Laboratory of Chemical Safety, College of Control Science and Engineering, China University of Petroleum (East China), Qingdao 266580, China; likangshuaiupc@163.com (K.L.); lina_zhou17@163.com (L.Z.); zhshy2020zhh@163.com (H.Z.); dzzhang@upc.edu.cn (D.Z.)

**Keywords:** triboelectric nanogenerators, conductive hydrogel, monitor human motion, wearable electronic devices

## Abstract

Wearable electronic devices have shown great application prospects in the fields of tactile sensing, electronic skin, and soft robots. However, the existing wearable electronic devices face limitations such as power supply challenges, lack of portability, and discomfort, which restrict their applications. The invention of triboelectric nanogenerators (TENGs) with dual functions of energy harvesting and sensing provides an innovative solution to address these issues. This study prepared a highly stretchable conductive hydrogel using doped conducting polymer poly(3,4-ethylenedioxythiophene):poly(styrene sulfonate) (PEDOT:PSS) as a strain sensor, demonstrating high sensitivity (GF = 4.31), an ultra-wide sensing range (0–1690%), ultra-fast response speed (0.15 s), excellent durability, and repeatability. A high-performance triboelectric nanogenerator was constructed using the hydrogel as an electrode, achieving an output performance of up to 192 V. Furthermore, the TENG fixed in the hands, wrists, legs, and feet of the human body can be used as a wearable electronic device to monitor human motion, which is conducive to promoting the development of triboelectric nanogenerators based on conductive hydrogels in strain sensors and self-powered wearable devices.

## 1. Introduction

With the rapid development of the Internet of Things (IoT) and advanced sensing technologies, portable, flexible, and wearable electronic devices are gaining widespread attention, especially in applications fields of electronic skin, soft robotics, and flexible transistors, showing tremendous potential [1,2,3,4]. However, most current wearable devices still rely on traditional external rigid power sources such as batteries or capacitors, which are not only faced with frequent charging or battery replacement, but also difficult to withstand drastic deformation, and fail to meet the requirements of lightweight and comfortable [5,6]. To address this issue, various self-powered devices have been reported, aiming to simplify the structure of wearable electronic devices, so that they can better fit the human skin or tissue without the need for external power [7,8,9,10,11]. Among these, triboelectric nanogenerators (TENGs) represent an innovative self-powering technology with dual functions of energy harvesting and sensing, and have been widely concerned because of their advantages of strong flexibility, simple structure, strong adaptability, and wide selection of materials [12,13,14,15,16]. The flexible TENG can be directly attached to the human body to convert the energy from human motion into electrical signals, which has potential application value in medical health monitoring, human movement recognition, and other fields [17,18,19,20]. However, achieving this goal requires that both the triboelectric layer and electrode of the TENG possess outstanding stretchability and flexibility. In terms of flexible triboelectric layers, a variety of nanomaterials such as polymers, rubber films, and textiles have been used as candidate materials [21,22,23,24]. However, the progress in the research of flexible electrodes or bionic skin electrodes is relatively limited. Although traditional flexible conductive materials, such as silver nanowires and carbon nanotubes, can be doped into elastomers to prepare flexible electrodes, they are prone to damage when subjected to stretching, twisting, bending, and other external forces, making it challenging to adapt to significant human body movements [25,26,27]. Therefore, the development of stretchable electrode materials for flexible TENGs holds crucial significance in driving the advancement of wearable electronic devices.

Hydrogels are emerging flexible electrode materials with a unique three-dimensional network structure, containing a large amount of water and a small number of hydrophilic macromolecules, capable of maintaining a solid state. Due to its excellent biocompatibility, stretchability, and expansibility, it is widely used in medical health, electronic skin, and sensors [28,29,30,31]. However, traditional hydrogels suffer limitations such as limited functionality, low conductivity, and lack of toughness, which restrict their applications. Interestingly, the chemical, mechanical, and electrochemical properties of hydrogels can be adjusted and improved by employing different functional crosslinkers and doping ions. Balancing the flexibility and conductivity of hydrogels can be achieved by introducing ions, nanoscale conductive materials, and conductive polymers as conductive media, providing hydrogels with new functionalities for multifaceted applications [32,33,34,35]. For example, Chowdhury et al. designed a dynamic redox process to synthesize CG/PAA/LS/Ni hydrogel using collagen, polyacrylic acid, lignosulfonate, and Ni^2+^, showing excellent flexibility and stretchability [36]. Conductive polymers, known for their tunable electrical properties and ease of synthesis, are typically used as fillers or main chains in conductive hydrogels, enhancing the electrical performance and sensitivity of hydrogels. This imparts the ability for conductive hydrogels to perceive the movement of dynamic objects, offering unique advantages in the field of wearable sensors [37,38,39,40].

In this study, a conductive hydrogel was synthesized by incorporating poly(3,4-ethylenedioxythiophene):poly(styrene sulfonate) (PEDOT:PSS) conductive polymer into a three-dimensional network hydrogel matrix based on acrylamide and gelatin. PEDOT enhanced the electrical conductivity of the hydrogel, while PSS improved the stability of PEDOT, providing the hydrogel with high sensitivity (GF = 4.31), an ultra-wide sensing range (0–1690%), ultra-fast response speed (0.15 s), excellent durability and repeatability. Furthermore, a stretchable triboelectric nanogenerator based on conductive hydrogel was developed to enable energy harvesting and human motion detection. In the contact–separation working mode, this TENG achieved an output voltage of up to 129 V, demonstrating excellent electrical performance. Undoubtedly, this advancement will propel the development of TENGs based on conductive hydrogels in the realms of strain sensors and self-powered wearable devices.

## 2. Materials and Methods

### 2.1. Materials

Gelatin, N, N’-methylenebisacrylamide (MBAA) were purchased from Shanghai Macklin Biochemical Reagent Co., Ltd., Shanghai, China. Acrylamide (AM), poly(3,4-ethylenedioxythiophene):poly(styrene sulfonate) (PEDOT:PSS), 2-hydroxy-4′-(2-hydroxyethoxy)-2-methylpropiophenone (Irgacure 2959) were purchased from Shanghai Aladdin Biochemical Technology Co., Ltd., Shanghai, China. Room temperature vulcanized silicone rubber was obtained from Smooth-On, Los Angeles, CA, USA. All reagents were used as received without further purification.

### 2.2. Preparation of PAM/Gelatin/PSS:PEDOT Hydrogel

The gelatin solution was obtained by mixing the gelatin into deionized water in a water bath at 60 °C. Acrylamide and crosslinking agent MBAA were added to the gelatin solution successively, and the gelatin solution was stirred until a uniform transparent solution was formed. The PEDOT:PSS suspension was injected into the transparent solution and stirred for 2 h. After the solution was evenly mixed, the photoinitiator 2-hydroxy-4′-(2-hydroxy-ethoxy)-2-methylphenylacetone was added and stirred for 1 h. After that, the solution is poured into the silicone mold and placed under ultraviolet light to initiate polymerization and complete the preparation of PAM/Gelatin/PSS:PEDOT (PGP) hydrogel. The PGP hydrogel strain sensor was obtained by connecting the two ends of the prepared PGP hydrogel with copper electrodes and fixing them with PI tape.

### 2.3. Preparation of PGP Triboelectric Nanogenerator

The curing agent of 4 wt% was added to the room temperature vulcanized silicone rubber, which was fully stirred and placed in the mold at room temperature for 48 h until the silicone rubber was completely cured and the triboelectric layer was obtained. The PGP-TENG adopted a sandwich structure, where the prepared PGP hydrogel (20 × 20 × 5 mm) was inserted between two layers of room-temperature vulcanized silicone rubber. The hydrogel was encapsulated by the adhesive room-temperature vulcanized silicone rubber to stabilize the structure of the TENG. Copper wires were connected to the hydrogel electrodes as leads for electrical signal measurements.

### 2.4. Characterization and Measurement

The surface morphology of PGP hydrogels was characterized by scanning electron microscopy (SEM, Hitachi-4800, Hitachi, Ltd., Tokyo, Japan). The functional group structure of PGP hydrogels was characterized by Fourier infrared spectroscopy (FTIR, Nexus 670, Thermo Nicolet Corporation, Madison, WI, USA). A tensile testing machine (TM2101-T5, Yigao Testing Machines Co., Ltd., Dongguan, China) was used to test the mechanical properties of PGP hydrogel. The PGP hydrogel strain sensor was fixed on the tensile testing machine for the tensile test, and the Agilent digital multimeter (Keysight 34470A, Keysight Technologies, Inc., Santa Rosa, CA, USA) was connected to the copper electrode of the PGP hydrogel strain sensor to obtain the sensing performance. The electrical output characteristics of PGP-TENG were tested by an Agilent digital multimeter (Keysight 34470A) and Agilent source/measurement unit (Keysight B2902A, Keysight Technologies, Inc., Santa Rosa, CA, USA).

## 3. Results

### 3.1. Design and Preparation of PGP Hydrogel

**Figure 1:**
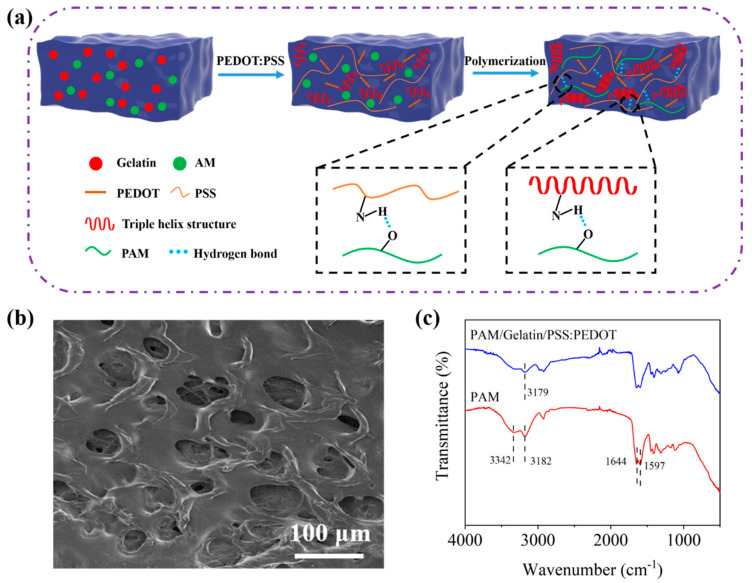
(**a**) Schematic of the preparation process for PAM/Gelatin/PSS:PEDOT hydrogel; (**b**) SEM image of the PGP hydrogel; (**c**) FTIR spectra of PAM hydrogel and PGP hydrogel.

Figure 1a shows the cross-linking mechanism of PGP conductive hydrogels, where acrylamide, gelatin, and randomly distributed PEDOT:PSS chains form a cross-linked PAM network. The SEM images of PGP hydrogels are shown in Figure 1b, exhibiting a distinct porous structure and revealing a polymer double network structure formed by PAM and PEDOT molecular chains. The uniform distribution of PEDOT:PSS generates well-connected conductive pathways, significantly enhancing the conductivity of the PGP hydrogel. The Fourier transform infrared (FTIR) spectrum of PGP hydrogel is shown in Figure 1c. In PAM hydrogel, the characteristic peak at 3182 cm^−1^ was the N-H stretching vibration, the absorption peak at 1652 cm^−1^ represented the carbonyl stretching vibration, and the absorption peak at 1609 cm^−1^ corresponded to the N-H bending vibration of the amide group. Compared with PAM hydrogels, there was a significant difference in the characteristic absorption peak curve of PGP hydrogels. The peak corresponding to N-H stretching vibration at 3182 cm^−1^ in PGP hydrogels was transferred to 3179 cm^−1^. This shift was attributed to the formation of hydrogen bonds between functional groups of Gelatin (such as -NH_2_, -OH, and -COO-) and -SO_3_- of PSS, as well as between PAM and PSS chains [41].

### 3.2. Mechanical Properties of PGP Hydrogel

**Figure 2:**
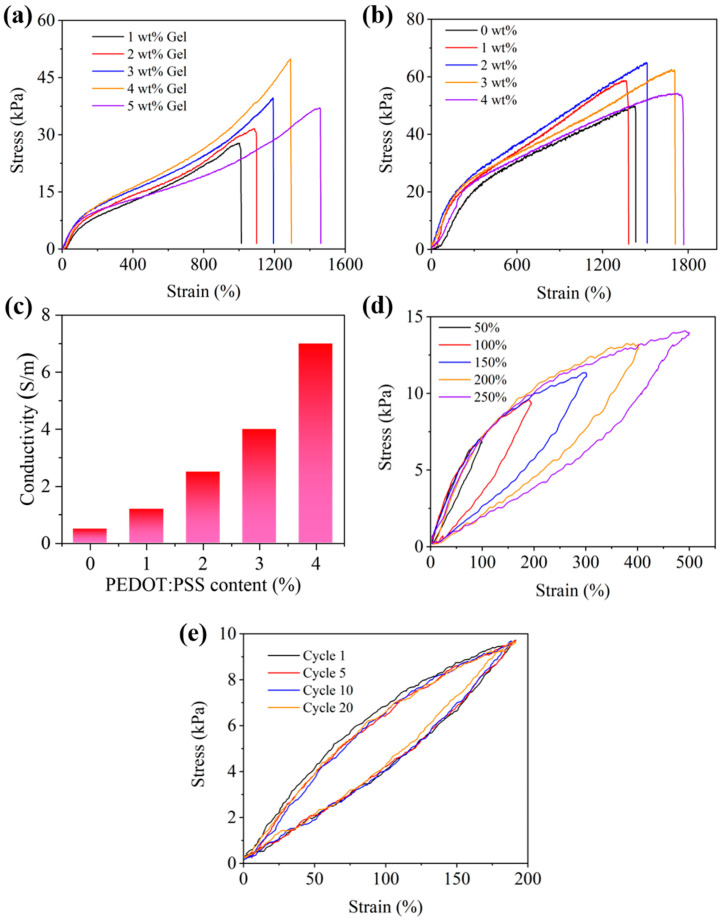
(**a**) The influence of Gelatin concentration on the tensile performance of the hydrogel; (**b**) the impact of PEDOT:PSS concentration on the tensile performance of the hydrogel; (**c**) conductivity of hydrogels with different PEDOT:PSS content; (**d**) PGP hydrogel under the different strain loading and unloading test; (**e**) under the same strain, PGP hydrogel loading and unloading test multiple times.

Wearable electronic devices must possess excellent mechanical properties to conform to the human skin or tissues. Stress and strain are crucial parameters for assessing mechanical performance. The influence of different gelatin contents on the tensile properties of PAM/Gelatin hydrogels was investigated. As shown in Figure 2a, with an increase in gelatin concentration from 1 wt% to 5 wt%, the elongation at break of the PAM/Gelatin hydrogel increased from 1140% to 1450%. This enhancement was attributed to the increase in hydrogen bonding within the hydrogel due to the higher gelatin content, thereby strengthening its tensile properties. Under the comprehensive consideration of strain length and strain strength, PAM/Gelatin hydrogel with gelatin content of 4 wt% was selected as the follow-up research object. Following the identification of the optimal gelatin concentration, the influence of PEDOT:PSS content on the tensile properties of PGP hydrogels was studied. As shown in Figure 2b, as the PEDOT:PSS content increased, both the elongation at break and tensile strength of the PGP hydrogel rose. This indicates that the introduction of an appropriate amount of PEDOT:PSS could effectively enhance the tensile strength and elongation at the break of the PGP hydrogel. This reinforcement effect is attributed to the ability of PEDOT:PSS to interact with PAM/gelatin through hydrogen bonding, thereby improving the mechanical properties of the hydrogel. The hydrogel exhibited optimal tensile properties when the PEDOT:PSS content reached 3 wt%, with an elongation at a break of 1690% and a high fracture strength of 62 kPa. The conductivity of hydrogels with different PEDOT:PSS contents were shown in Figure 2c, when the concentration of PEDOT:PSS in hydrogels was 0 wt%, the conductivity was low. After adding 1 wt% PEDOT:PSS, the conductivity increased by three times, and with the increase in PEDOT:PSS content, the conductivity of hydrogel also increased gradually.

Fatigue performance is also an important characteristic for assessing mechanical properties. To investigate the fatigue performance of the PGP hydrogel, a series of cyclic loading and unloading tests were conducted to study the energy dissipation process and toughness mechanism of the PGP hydrogel. During deformation, dynamic non-covalent bonds in the dual-network structure were prone to fracture. These hydrogen bonds acted as reversible “sacrificial bonds” that participated in energy dissipation during dissociation and reconstruction processes, enabling the PGP hydrogel to exhibit high toughness and excellent recovery properties. As shown in Figure 2d, as the tensile strain increased from 50% to 250%, the cyclic tensile loading–unloading curve of the hydrogel exhibited hysteresis loops and residual strain. Moreover, the area of hysteresis increased with the strain, indicating a significant increase in the dissipated energy of the hydrogel. This suggested that the PGP hydrogel possessed efficient energy dissipation capabilities. Figure 2e illustrates the durability test of the PGP hydrogel, where during continuous cyclic loading and unloading processes of the same length, the tensile strength of the hydrogel remained nearly unchanged, staying at 95% of its initial value. These results demonstrated the remarkable structural stability of the PGP hydrogel, which provided strong support for its potential use as a strain sensor in the future.

### 3.3. PGP Hydrogel Strain Sensor

**Figure 3:**
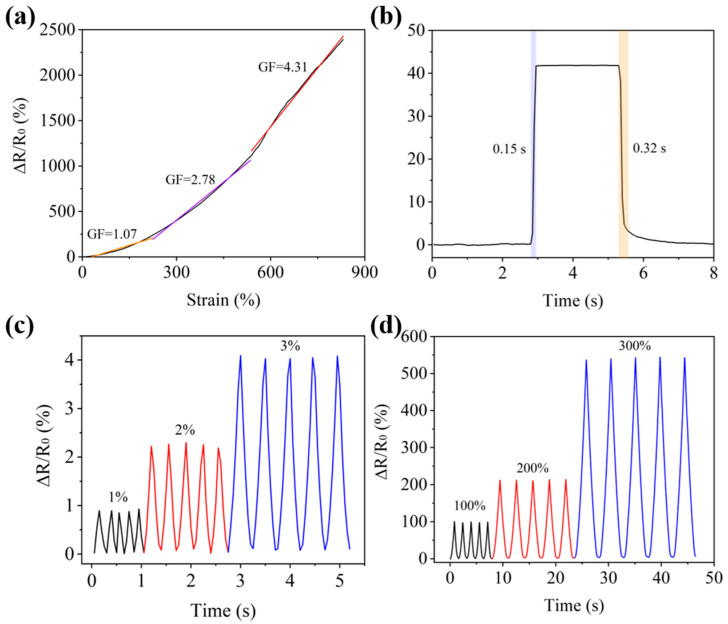
(**a**) Strain coefficient of the PGP hydrogel strain sensor. (**b**) Response recovery time of the PGP hydrogel strain sensor. (**c**) The response of PGP hydrogel strain sensors under small strains of 1%, 2%, and 3%. (**d**) The response of PGP hydrogel strain sensors under large strains of 100%, 200%, and 300%.

The PGP hydrogel exhibited excellent flexibility and could serve as a strain sensor. The performance of the hydrogel strain sensor was evaluated through resistive strain sensing. The equation of the resistance change rate is ∆R/R_0_ = (R − R_0_)/R_0_, where R is the real-time resistance, R_0_ is the initial resistance, and ∆R represents the change in resistance. The sensitivity parameter of the PGP hydrogel is defined by the gauge factor (GF), where GF = (∆R/R_0_)/ε, with ε representing the strain on the PGP hydrogel sensor. As shown in Figure 3a, the ΔR/R_0_ of the PGP hydrogel strain sensor during mechanical deformation was tested. The relative resistance of the PGP hydrogel strain sensor increased with the extension length and exhibited three linear response regions, namely 0–220% (GF = 1.07), 100–530% (GF = 2.78), and above 530% (GF = 4.31). It is evident that the PGP hydrogel strain sensor demonstrated excellent responsiveness to variations in different extension lengths. Figure 3b shows the response and recovery times of the PGP hydrogel strain sensor. The sensor exhibited a rapid response to strain, with response and recovery times of 0.15 s and 0.32 s, respectively. This indicates that the PGP hydrogel strain sensor possessed highly sensitive response characteristics and showed no significant delay during the response process. Furthermore, to investigate the response–recovery stability of the PGP hydrogel strain sensor, the sensor was subjected to multiple strain tests under low strain (1%, 2%, 3%) and high strain (100%, 200%, 300%) conditions. As shown in Figure 3c,d, the response signals for different strains exhibited linear changes. The reason was that with the increase in strain, the conductive path of the sensor changes, resulting in a change in response. Additionally, the relative resistance changes exhibited good repeatability under the same tensile strain conditions, which indicated that the sensor has high stability and reliability. As shown in Figure 3c,d, when the hydrogel was gradually stretched from 0% to 300%, the ΔR/R_0_ response strength increased with the increase in strain amplitude, and the response value was basically unchanged during repeated stretching, indicating that the hydrogel had good stability and response consistency. At the same time, hydrogels showed different strength responses to different strains, which indicated that hydrogels could stably detect and distinguish different strains.

**Figure 4:**
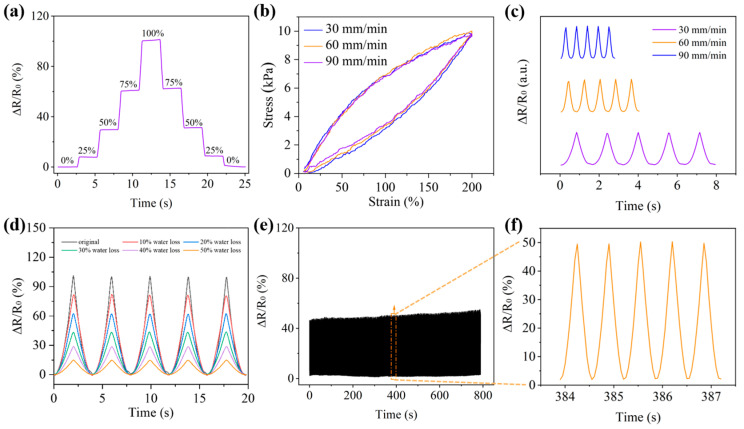
(**a**) The step response of PGP hydrogel strain sensors. (**b**) Cyclic loading and unloading tests of hydrogel strain sensors at different tensile rates. (**c**) The response of strain sensors at different tensile rates. (**d**) The response of hydrogels with different degrees of water loss under 100% stretching conditions. (**e**) The response of strain sensors under long-term working conditions. (**f**) Amplified diagram of long-term working response.

The step-wise strain responses of the PGP hydrogel strain sensor at 0%, 25%, 50%, 75%, and 100% elongation were illustrated in Figure 4a. The responses could reliably return to the initial values, showcasing excellent strain-induced stability and reversibility. Under 100% strain conditions, hydrogel cyclic loading and unloading experiments were carried out at different rates. As shown in Figure 4b, there was little change in energy dissipation during the loading and unloading of the hydrogel. The response of the strain sensor under the same strain size but at different stretching speeds is shown in Figure 4c. While the response magnitude remained consistent, the response time showed a negative correlation with stretching speed. As shown in Figure 4d, the water content of the hydrogel affected the strain-sensing performance. As the water content decreased, the response of the hydrogel strain sensor decreased. Under 100% stretching conditions, when the water content of the hydrogel was less than 50%, the sensor could not undergo stretching. Therefore, to ensure the stable performance of the sensor, a silicone rubber coating was applied on the surface of the hydrogel to reduce water loss. As shown in Figure 4e, durability and repeatability tests of the PGP hydrogel sensor were conducted through continuous tensile experiments. During long-term stretching and recovery cycles, the response of the hydrogel sensor remained stable. From the magnified view in Figure 4f, the response signal exhibited linear changes during each strain cycle. This indicated that the strain sensor could maintain a relatively stable response over extended periods of operation, and this advantage could be extended in subsequent practical applications to ensure that the sensor maintains a stable working state during use.

### 3.4. TENG Based on PGP Hydrogel

**Figure 5:**
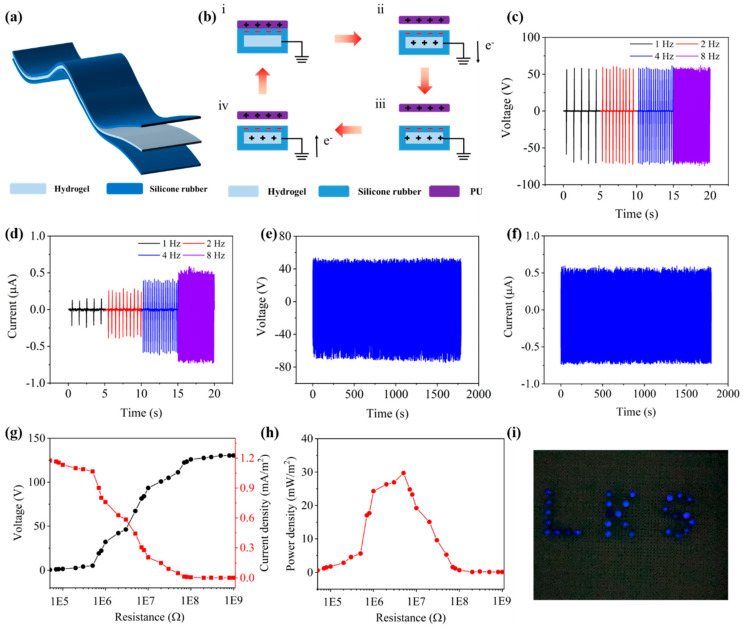
(**a**) Schematic diagram of the PGP-TENG. (**b**) Working principle diagram of the PGP-TENG. (**c**) The output voltage of the PGP-TENG under different contact–separation frequencies. (**d**) The output current of the PGP-TENG under different contact–separation frequencies. (**e**) Long-term output voltage of the PGP-TENG. (**f**) Long-term output current of PGP-TENG. (**g**) Voltage and current of the PGP-TENG under different loads. (**h**) Power density of PGP-TENG under different loads. (**i**) The PGP-TENG used for capacitor charging.

The model of the PGP-TENG is shown in Figure 5a; both the PGP hydrogel electrode and the silicone rubber triboelectric layer were stretchable. The working principle of the PGP-TENG is shown in Figure 5b. When polyurethane (PU) came into contact with the PGP-TENG, due to contact electrification and electrostatic induction effects, the silicone rubber became negatively charged, while the PU became positively charged simultaneously. When the triboelectric layer was separated, an electrostatic potential difference was created, leading to the redistribution of ions within the hydrogel. The attraction of negative charges on the silicone rubber caused the positive charges in the hydrogel to be drawn towards it. Electrons flowed out through the grounded external circuit, inducing transient current flow. As the positively charged PU moved back towards the PGP-TENG, the electrostatic potential difference was neutralized, causing electrons to be attracted back through the grounded external circuit, resulting in another transient current flowing in the opposite direction. This repetitive cycle generates a continuous alternating current. To measure the output performance and energy harvesting capabilities of the PGP-TENG, polyurethane (PU) was selected as the contact material for systematic testing of the PGP-TENG. Figure 5c shows the relationship between the output performance of the PGP-TENG and frequency. The output voltage of the TENG was tested at frequencies of 1, 2, 4, and 8 Hz. The results indicated that the open-circuit voltage of the PGP-TENG showed no significant correlation with the frequency of contact separation. In other words, the output voltage of the TENG remained consistent at different operating frequencies. However, as shown in Figure 5d, the current of the PGP-TENG increased proportionally with frequency. This is due to the increase in current with frequency, as each contact–separation cycle per unit of time enhanced the contact–separation rate between the triboelectric layers, thereby increasing the speed of charge transfer on the hydrogel electrode.

The stability and durability of the TENG are the key properties to determine the practical application; therefore, the durability of the PGP-TENG was tested. As shown in Figure 5e,f, under a fixed contact–separation frequency, the open circuit voltage of the PGP-TENG could be stabilized at about 129 V after a 30 min cycle, with both the voltage and current amplitudes remaining constant. This indicates the device has stability and long-term reliability. Studying the output performance of the TENG under different load resistances is crucial for practical applications. Figure 5g presents the testing of the TENG’s output voltage and output current density under various load resistances. As the load resistance increases from 5 × 10^4^ Ω to 1 × 10^9^ Ω, the output voltage increases accordingly, while the current density exhibits an opposite trend, decreasing as the load resistance increases. The power density diagram was obtained according to the power calculation formula P = U^2^/RA, where A represents the contact area, as shown in Figure 5h. The output power initially increased and then decreased, and the maximum output power density was 30.1 mW/m^2^. TENGs were commonly used as nanoscale power sources, harvesting tiny mechanical energy such as a human motion to power small electronic devices like LED lights. The PGP-TENG was used to power the LED light in the contact–separation mode. As shown in Figure 5i, the LED light was successfully lit by the TENG. By comparing with recently published research, the PGP-TENG has significant advantages in terms of sensitivity and output voltage, and the specific results are shown in Table 1.

**Figure 6:**
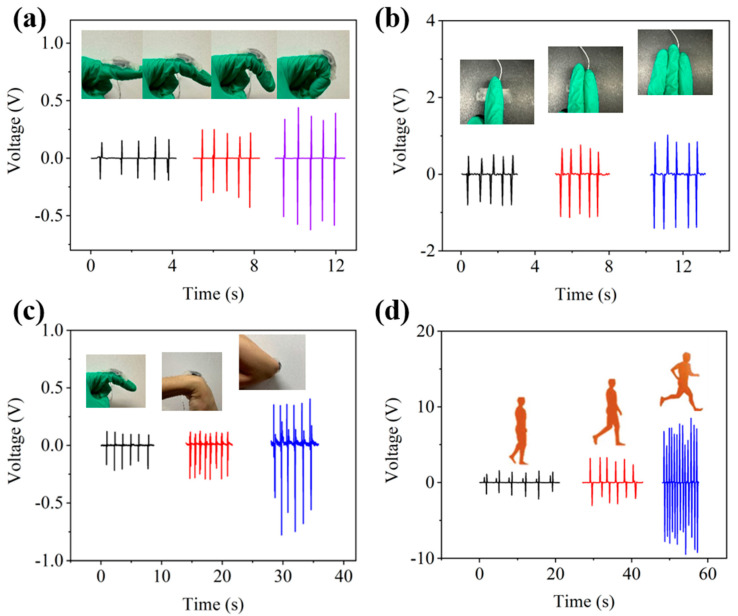
(**a**) Schematic diagram of the PGP-TENG. (**b**) PGP-TENG detects the number of finger taps. (**c**) PGP-TENG detects the movement of fingers, wrists, and elbows. (**d**) PGP-TENG detects the walking and running status of the human body.

The PGP-TENG could be used as a self-powered sensor to detect human motion. The PGP-TENG was fixed at the knuckle of the finger, and the bending and straightening of the finger caused the contact separation between the human body and PGP-TENG. As shown in Figure 6a, the PGP-TENG could effectively track the continuous bending movements of the finger and represent them through changes in electrical signals. As the bending angle of the finger increased gradually from 0° to 90°, the output voltage of the PGP-TENG sensor also rose from 0.4 V to 1.2 V, demonstrating its high sensitivity in capturing subtle bends. The voltage variation was a result of the increased contact area between the finger and the triboelectric layer of the TENG as the finger bends, leading to a higher output voltage from the PGP-TENG sensor. At each specific angle, the output signal of the PGP-TENG showed good repeatability, indicating the reliability and consistency of the self-powered wearable sensor. Furthermore, the PGP-TENG could harness mechanical energy from human motion. When fixed on a desktop for tapping tests, as shown in Figure 6b, the voltage magnitude increased with the number of fingers used for tapping. This is because as the number of fingers increased, the contact area between the human body and the PGP-TENG also increased.

The PGP-TENG could detect motion variations in different parts of the human body. The PGP-TENG was attached to the finger, wrist, and elbow, respectively, and the bending movement was tested. As shown in Figure 6c, there is little difference in the amplitude of the voltage when the finger and wrist were bent. However, when the elbow was bent, the voltage significantly increased due to the substantial change in the contact–separation area. In the field of motion detection, the PGP-TENG could also be utilized to monitor the state of human walking and running. The PGP-TENG was attached to the foot, and the socks acted as the triboelectric layer to carry out slow walking, fast walking, and running, respectively. The voltage signals generated are shown in Figure 6d. With the increase in movement frequency and intensity, the voltage signal gradually increased, which was due to the increase in movement speed during running, and the signal generated by running was significantly higher than the signal generated by walking. Therefore, depending on the frequency and pressure changes in human movement, PGP-TENG could generate a variety of voltage signals. These signals could distinguish the different motion states of the human body and provide a new monitoring method for motion monitoring.

## 4. Conclusions

In summary, the polymer conductive hydrogels prepared based on PAM/Gelatin/PSS:PEDOT exhibited high stretchability and stability. The strain sensor fabricated using hydrogel demonstrated a rapid and stable response to mechanical strain, with a sensitivity of 4.31 and a response recovery time of 0.15 s and 0.32 s, respectively. PGP hydrogel was assembled as an electrode to form the PGP-TENG, and its energy harvesting performance and human motion detection performance were tested. In the contact–separation operating mode, the PGP-TENG could output a voltage of 129 V. The human body was rich in various forms of mechanical energy, and the PGP-TENG was fixed on the hand, wrist, leg, and foot of the human body, respectively, as a wearable TENG to collect biological mechanical energy. When the human body moves, the PGP-TENG and the human body surface generate contact separation to generate stable voltage signals. This work aims to expand the application of TENGs based on conductive hydrogel in the field of human motion detection and biological health, but there are still some problems to be solved, which need to be improved through more in-depth research work. At present, most hydrogels are mainly used as electrodes for TENGs, but the configuration of TENGs is relatively simple, and the single-electrode mode is usually used, resulting in a low power density. Further research and development are necessary to create more diverse operating modes for hydrogel-based TENGs to increase output power and expand application areas. In addition, in future application research, especially in the fields of brain–computer interface, biosensor, and implantable medical treatment, TENGs have a wide range of application prospects, so it is necessary to further study the interaction between the TENG and the human body, biocompatibility, and fully biodegradable properties.

## Figures and Tables

**Table 1:** Comparison of the performance between the reported hydrogel-based flexible triboelectric nanogenerator and this work.

Hydrogel Material	Triboelectric Layer Material	Elongation at Break/%	Power Density/(mW·m^−2^)	Ref.
PAM/NaOH/lignin	VHB/Copper	640	29.6	[42]
HECM/PAA/PPy	PDMS/PU	316.2	13.6	[43]
PAA/CS/MWA/SBMA/LiCl	Ecoflex/Kapton	210	7.04	[44]
PEGDA/Laponite XLS	Ecoflex/Skin	1120	23.2	[45]
AM/Gelatin/PSS: PEDOT	Rubber/PU	1690	30.1	This work

## Data Availability

The original contributions presented in this study are included in the article. Further inquiries can be directed to the corresponding authors.
